# Functional 5′ UTR motif discovery with LESMoN: Local Enrichment of Sequence Motifs in biological Networks

**DOI:** 10.1093/nar/gkx751

**Published:** 2017-08-31

**Authors:** Mathieu Lavallée-Adam, Philippe Cloutier, Benoit Coulombe, Mathieu Blanchette

**Affiliations:** 1 McGill Centre for Bioinformatics and School of Computer Science, McGill University, Montréal, Québec H3A 0E9, Canada; 2 Ottawa Institute of Systems Biology and Department of Biochemistry, Microbiology and Immunology, Faculty of Medicine, University of Ottawa, Ottawa, Ontario K1H 8M5, Canada; 3 Translational Proteomics Laboratory, Institut de recherches cliniques de Montréal, Montréal, Québec H2W 1R7, Canada; 4 Département de biochimie et médecine moléculaire, Université de Montréal, Montréal, Québec H3C 3J7, Canada

## Abstract

Biological networks are rich representations of the relationships between entities such as genes or proteins and have become increasingly complete thanks to various high-throughput network mapping experimental approaches. Here, we propose a method to use such networks to guide the search for functional sequence motifs. Specifically, we introduce Local Enrichment of Sequence Motifs in biological Networks (LESMoN), an enumerative motif discovery algorithm that identifies 5′ untranslated region (UTR) sequence motifs whose associated proteins form unexpectedly dense clusters in a given biological network. When applied to the human protein–protein interaction network from BioGRID, LESMoN identifies several highly significant 5′ UTR sequence motifs, including both previously known motifs and uncharacterized ones. The vast majority of these motifs are evolutionary conserved and the genes containing them are significantly enriched for various gene ontology terms suggesting new associations between 5′ UTR motifs and a number of biological processes. We validate *in vivo* the role in protein expression regulation of three motifs identified by LESMoN.

## INTRODUCTION

**Figure 1.**
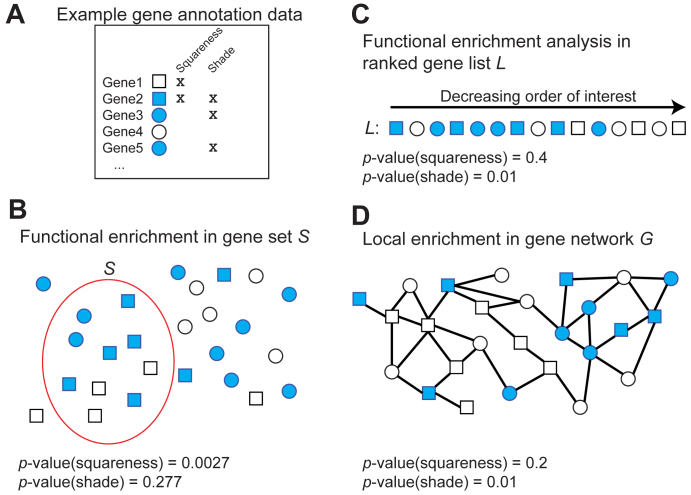
Examples of various notions of enrichment. (**A**) Each marker is a gene, which can has two properties: squareness and/or shade. (**B**) A set *S* of interest (e.g. differentially expressed under some experimental condition) is enriched for the squareness property but not the shade property. (**C**) In a ranked list of genes *L* (e.g. based on the degree of differential expression), the shade property is enriched at the top of the list. (**D**) In a gene network *G*, the shade property is locally enriched in the top-right portion of the graph.

Gene set enrichment analyses, where one identifies properties that are found in a set of genes of interest more often than expected by chance, are some of the most powerful and commonly used approaches for the analysis of large biological datasets. Here, the set of genes of interest may correspond to those that are differentially expressed between experimental conditions, cell types or diseases, targeted by a given transcription factor or miRNA or encoding a set of interacting proteins. The properties or annotations, considered may originate from the functional annotations of the gene ontology (GO) project (1), pathway databases such as KEGG (2), disease-associations provided in the Online Mendelian Inheritance in Man repository (3) or more comprehensively from the Molecular Signature Database (MSigDB) (4). However, more generally, any mathematical function that separates genes into two sets–those that possess the property and those that do not–can be used for gene set enrichment analysis (see Figure 1A and B for an example). Irrespective of the nature of the property considered, an enrichment for a given property suggests a direct or indirect relationship between that property and the set of genes, provided appropriate controls and statistical approaches are used. A typical strategy to test for the enrichment of a property (e.g. originating from GO or MSigDB) in a given set of genes $S$ taken from the whole set of genes ${\rm{\Omega }}$ of an organism is to perform a hypergeometric or Fisher’s exact test (5–8).

The type of gene sets used as input for the above-mentioned analyses can be characterized as ‘unstructured’, since each gene they contain contributes equally to the enrichment analysis (9). An extension of such a strategy was explored by the Gene Set Enrichment Analysis (GSEA) computational tool (10) and a number of related approaches (11–15). Instead of separating genes into those that are ‘of interest’ and those that are not and seeking properties that are enriched in the former, GSEA takes as input a ranked list of genes based on their ‘level of interest’ with respect to a particular measure (e.g. over-expression in a given condition) and identifies properties whose distribution in the ranked list is non-uniform (Figure 1C). In that sense, we could say that GSEA takes advantage of a ‘weak structure’ defined on ${\rm{\Omega }}$ by the measure of interest, to identify annotations that are non-randomly distributed in this structured space.

In previous work, we developed GoNet, a GO enrichment analysis that can be applied to much richer structures such as those defined by biological networks, e.g. protein–protein interaction (PPI) networks (9). In that case, properties (GO terms) of interest were those where the genes (or proteins) with the property were non-randomly distributed in the network, i.e. more clustered than expected by chance, representing the so-called local enrichment of the property (Figure 1D).

Although not typically presented this way, sequence motif discovery algorithms such as expectation-maximization algorithms (MEME (16)), Gibbs sampling (AlignACE (17)), word statistics approaches (YMF (18)) or ensemble approaches (SeSiMCMC (19), Amadeus (20)) also fall under the umbrella of enrichment analysis. Here, the properties of interest are the presence/absence of a particular sequence motif in a gene’s sequence, its regulatory regions or the protein it encodes and one seeks motifs that are enriched in a given set of genes compared to a control set. The idea behind GSEA was also used to generalize motif discovery approaches, where one now considers an ordered list of sequences (ranked by differential expression, binding affinity to a given transcription factor or other relevant measures) and identifies motifs that are unevenly distributed in the list (21–24). Another group recently identified RNA regulatory elements that are involved in the gene regulatory network of *Trypanosoma brucei* using a graph-based approach (25).

In this paper, we introduce the Local Enrichment of Sequence Motifs in biological Networks (LESMoN) approach, a sequence motif discovery approach that is guided by a biological network. Given a biological network (here, a PPI network) with sequences associated to each node, LESMoN identifies short sequence motifs whose containing sequences are unexpectedly clustered in the network, i.e. locally enriched motifs.

We use LESMoN to identify functional sequence motifs found in genes’ 5′ untranslated regions (UTRs), which are sequences that play key roles in post-transcriptional regulation. Specific 5′ UTR primary and secondary structure motifs regulate translation (26–30). For example, the 5′ UTRs of ribosomal genes and other genes involved in protein synthesis often contain a 5′ TOP motif (31,32) that regulates translation initiation of mRNAs (33). Furthermore, 5′ UTRs often contain intracellular localization elements, which are required for the binding of their mRNAs to certain cell structures such as membranes (34) and synapses (35). DNA that encodes 5′ UTRs can also harbor transcriptional regulatory regions such as transcription factor-binding sites. Consequently, computational approaches that improve our understanding of motifs located in UTRs, such as the comparative genomics approach proposed by Xie *et al.* (36), are likely to be valuable to better understand both transcriptional and post-transcriptional regulation.

As we show in this paper, LESMoN is capable of identifying a large set of sequence motifs that associate with specific functional subnetworks, including motifs involved in transcription, translation, splicing, cell cycle processes and others. LESMoN identified more 5′UTR motifs than GoNet and a conventional motif discovery approach. Motifs identified by LESMoN include both previously known functional motifs (e.g. 5′ TOP motifs), as well as currently uncharacterized ones. Additional evidence (inter-species conservation, position, strand biases, GO enrichment of corresponding proteins, etc.) points to specific functions for most motifs. We validate the functional role of some of the motifs identified by LESMoN *in vivo*. All motifs tested showed a significant protein expression response upon their mutation.

## MATERIALS AND METHODS

### Method overview

The goal of our approach is to find 5′ UTR sequence motifs for which the associated protein products exhibit a higher degree of clustering in a given PPI network than what would be expected by chance. We enumerate all possible motifs of a given length and over a given alphabet (described below) and test whether the sequences that contain the motif are clustered in the network. To this end, we present a measure of the degree of clustering of a subset of nodes in a network and propose efficient algorithms to evaluate the statistical significance of that clustering. Should the proteins associated with a given motif be significantly clustered, this would suggest that the motif is linked directly or indirectly to the biological mechanism causing the clustering of the associated proteins in the PPI network. We also present strategies to evaluate the biological significance of the motifs identified by the above-mentioned approach.

### Protein–protein interaction network

We tested our approach on the human PPI network downloaded from the BioGRID database (version 3.2.97) (37,38), one of the most comprehensive human PPI network available. The network contains 14 113 proteins forming 127 433 unique pairwise interactions. Even if this network can be treated as directed because of the nature of some of the experiments used to build it (e.g. affinity purification involving a bait and prey), we consider it as undirected since edge directionality is only an artefact of experimental methods and is generally irrelevant when considering the real biological data in this context. We extracted the largest connected component of that network and removed four proteins (CUL3, SUMO2, ELAVL1 and UBC) with an exceedingly large number of interactions (>1000), as those negatively affect LESMoN’s performances by connecting proteins that are for the most part unrelated. The resulting network $G\ = \ ( {V,E} )$ contains $| V | = \ 12 133$ proteins and $| E | = \ 94 490$ interactions.

### 5′ UTR motif enumeration

5′ UTR sequences of the mRNAs encoding proteins present in the human BioGRID PPI network were obtained from the RefSeq gene annotation database through the UCSC Table Browser (28 Feburary 2013). When a protein was associated with multiple 5′ UTR variants, their union was associated with that protein. To avoid issues related to incomplete or inaccurate annotations of start codons of some transcripts, we only considered for each 5′ UTR the first 500 nts (at most) downstream of the transcription start site (TSS). This includes the full length of >90% of 5′ UTRs in our dataset. We then enumerated sequence motifs of length 8 over the nucleotide alphabet $\mathop \sum \nolimits = \ \ \{ {A,\ C,\ G,\ U,\ R,\ Y,\ N} \}$, where $R\ = \ A|G$, $Y\ = \ C|U$ and $N\ = \ A| C |G|U$. A protein was annotated as containing a given motif if the corresponding 5′ UTR had at least one match to that motif, considering only the forward strand (i.e. matches to the reverse complement sequence were not considered). Evaluating the statistical significance of the clustering of motifs that are associated to >1500 proteins would require a large amount of computational time. LESMoN therefore ignores such motifs that are likely to contain several degenerate characters and to be of limited biological interest.

### Clustering measure

We used the Floyd–Warshall’s algorithm (39,40) to calculate the distance matrix ${d_G}$ defined by $G$, where ${d_G}( {u,v} )$ is the length of the shortest path in $G$ between nodes $u$ and $v$. Now let ${V_m} \subseteq V$ be the set of all proteins annotated with the motif $m$. We previously defined a distance measure for the proteins in ${V_m}$ called the total pairwise distance (TPD), defined as the sum of all pairwise distances of the proteins in ${V_m}$ (9). This measure is however sensitive to outliers. For instance, if ${V_m}$ consists of a group of proteins that are tightly clustered but also other proteins that are at a large distance of the clustered group, then the TPD of ${V_m}$ will be fairly large. In addition, not all occurrences of a given motif are expected to be functional (some may simply occur by chance), therefore we also do not expect all occurrences of a motif to be clustered in the network. Hence, we propose an alternative measure, called the top percent pairwise distance (TPPD), that accounts for this situation and focuses only on the proteins in ${V_m}$ that are the most clustered. Let $N_m^l( u ) \subseteq {V_m} \setminus \{ u \}$ be the set of $l$ closest nodes from $u$ in ${V_m}$ and define $D_m^l(u) = \sum\nolimits_{v \in N_m^l(u)} {{d_G}(u,v)}$. In other words, $D_m^l( u )$ is the sum of the $l$ smallest distances between $u$ and other proteins that are annotated with $m$. Define *core*(${V_m}$)$\ \subseteq {V_m}$ as the subset of $l$ nodes of ${V_m}$ for which the $D_m^l$ values are the smallest and let $T_m^l = \ \mathop \sum \nolimits_{u \in core( {{V_m}} )} D_m^l( u )$. Then, if ${V_m}$ contains a tight cluster of size $l$, it will correspond to *core*(${V_m}$) and $T_m^l$ will be small. Empirical investigations suggested that choosing $l = 0.1 \cdot |{V_m}|$ (top 10%) yields the most high-confidence results over $l = 0.05 \cdot |{V_m}|$ (top 5%) and $l = 0.2 \cdot |{V_m}|$ (top 20%). We thus used $TPPD({V_m}) = T_m^{0.1 \cdot |{V_m}|}$ to identify 5′ UTR motifs in the BioGRID network.

### Clustering statistical significance

We previously showed how to evaluate the statistical significance of $TPD({V_m})$ for a given ${V_m}$ and a given network (9). However, that approach only works for small sets of proteins ($| {{V_m}} | < 100$), uses a null model that is not appropriate here and cannot be easily extended to the TPPD. The approach presented here is therefore slightly different. This strategy computes the distribution of the random variable ${S_k} = \ TPPD($R$)$, where R $ = \{ {{r_1},{r_2}, \ldots ,\ {r_k}} \}\ \subseteq \{ {1, \ldots ,| V |} \}$ is a randomly selected subset of $k$ proteins. Contrary to our previous work where every node in the network was chosen to be part of $R$ with equal probability, a more appropriate null model is one where the probability that a given protein is selected in ${S_k}$ is proportional to the length of its 5′ UTR. To evaluate the statistical significance of the clustering of the proteins associated with a motif $m$, a $P$-value is then calculated as follows: *P*-value $( m ) = \Pr [ {{S_{| {{V_m}} |}} \le TTPD( {{V_m}} )} ]$. In order to compute clustering *P*-values, we introduce two methods to approximate the distribution of ${S_k}$, one for protein sets with small cardinality ($|{V_m}| \le 300$) and another for larger sets ($|{V_m}|$ > 300).

### Monte Carlo sampling

We showed previously that the exact computation of the distribution of ${S_k}$ with the TPD distance measure is NP-hard (9). Since the TPPD is a generalization of the TPD, the same complexity result carries. We therefore cannot expect to perform this calculation exactly in polynomial time. Nevertheless, the statistical significance of the level of clustering of a set of proteins can be estimated using Monte Carlo sampling, where $k$ proteins are repeatedly sampled and the TPPD evaluated in order to estimate the distribution of ${S_k}$. Because the time required to compute TPPD(${S_k}$) is O$({k^2}$) in the worst case (once the full pairwise distance matrix ${d_G}$ is computed) and this procedure needs to be repeated a large number of times (e.g. 10^6^ times to obtain a *P*-value accuracy of ∼10^−6^), it is only reasonably feasible for values of $k$$ \le$ 300. However, for most motifs $m$, $| {{V_m}} | >300$, so a faster approach is required.

### Normal approximation

We previously demonstrated that the distribution of ${S_k}$ for TPD can be estimated using a normal distribution when $k$ and $| V |$ are large (9). We therefore propose to estimate the distribution of ${S_k}$ when $k >300$ with a normal distribution $\mathcal{N}( {{\mu _k},\sigma _k^2} )$. For each value of $k$ between 301 and 1500, we estimate ${\mu _k}$ and $\sigma _k^2$ using Monte Carlo sampling (sample size 10^5^). The estimated normal distributions are then used to obtain the desired *P*-values. The significance of the clustering of motifs present in >1500 5′ UTRs is not assessed due to the excessive computational burden. This does not represent a big loss, as these motifs are likely to be largely composed of degenerate characters (R, Y and N) and to have very little biological significance. We also use this normal approximation for cases where $k \le 300$ and where the *P*-value estimated by the full Monte Carlo sampling from the previous section is too small to be estimated accurately (<10^−6^; i.e. none of the 1 000 000 random samples had a TPPD $ \le$$TPPD( {{V_m}} )$).

### False discovery rate calculation

Since a large number of 5′ UTR motifs (at most 7^8^$ \approx$ 5.8 millions) are tested for the clustering significance of their associated proteins, multiple hypothesis testing is a significant issue. These statistical tests are far from being independent, since many motifs tested are variants of each other, making a *P*-value correction such as a Bonferroni correction (41) overly stringent. To address this issue, we scrambled the 5′ UTR sequences in our dataset to estimate a false discovery rate (FDR) for any given clustering *P*-value threshold. More precisely, the order of the nucleotides of each 5′ UTR sequence is permuted within non-overlapping windows of 10 nts, in order to preserve local sequence properties such as GC content. Motif clustering *P*-values are then obtained for this scrambled dataset, using the same procedure as described above. Let $M( p )$ be the number of motifs that obtained a *P*-value at most $p$ in the actual set of sequences and $N( p )$ be the number of such motifs in the scrambled dataset. We then calculate the FDR for a given *P*-value $p$ as $FDR\ ( p ) = \ N( p )/M( p )$.

### Grouping 5′ UTR motifs into families

To facilitate the analysis and reduce the redundancy of the motifs LESMoN detected, we used a hierarchical clustering approach to group similar motifs into families based on the overlap of the sets of proteins they are associated with. Specifically, let ${m_1}$ and ${m_2}$ be two motifs and ${V_{{m_1}}}$ and ${V_{{m_2}}}$ be their associated sets of proteins. We define the similarity between ${m_1}$ and ${m_2}$ as

\begin{equation*}s\ \left( {{m_1},{m_2}} \right) = \frac{{\left| {{V_{{m_1}}}\mathop \cap \nolimits \, {V_{{m_2}}}} \right|{\rm{\ }}}}{{{\rm{min}}\left( {\left| {{V_{{m_1}}}} \right|,\left| {{V_{{m_2}}}} \right|} \right)}}\ \end{equation*}

and turn this into a distance measure $d\ ( {{m_1},{m_2}} ) = 1/s(\ {m_1},{m_2}) - 1$.

A hierarchical clustering tree is then constructed using the average linkage algorithm (42) (using the ‘cluster’ R package (43)) with this distance measure. The resulting tree is displayed using the A2R R package: (http://addictedtor.free.fr/Download/A2R.zip). A cut in the tree is performed to identify a reasonable number of motif families (200), each of which can be represented using a Weblogo (44). The motif that obtained the best clustering *P*-value in a given family is selected as the representative member of the family.

### Benchmark with conventional motif discovery tool

Since there is no single computational method that performs the same analysis that LESMoN executes, we benchmarked our method against two state-of-the-art tools that, when combined together, identify sequence motifs that are clustered in PPI networks. The Markov Clustering algorithm (MCL) (http://micans.org/mcl/) (45,46) was first used to identify protein clusters, using the recommended inflation parameter value of 2. The Multiple EM for Motif Elicitation (MEME) (47) software package was then run to detect motifs of length 8 that would be over-represented among the 5′ UTR of the genes in these clusters (*E*-value < 1). To the best of our knowledge, the coupling of these two methods was the closest approach to LESMoN at the time of writing this paper. Also, for each cluster, the locally randomized 5′ UTR sequences of the same proteins were also submitted to MEME to estimate the FDR of this approach as the ratio of the number of motifs with an *E*-value < 1 in locally randomized sequences and the number of motifs with an *E*-value < 1 in real 5′ UTR sequences.

### 5′ UTR motif conservation

To further explore the biological significance of the motifs detected by LESMoN, we evaluate their level of evolutionary conservation. For each motif $m$, we compute the fraction $Cons( m )$ of motif occurrences in *core*(${V_m}$) whose middle position is contained within a highly conserved genomic region among placentals (phastConsElements46wayPlacental (48) from the UCSC Genome Browser). We then compute the conservation fold-enrichment $( m )\ = \ Cons( m )/Cons( * )$, where $Cons( * )$ is the fraction of all human 5′ UTR bases located in conserved regions.

### Gene ontology enrichment analysis

To investigate the mechanisms in which the significantly clustered motifs identified by LESMoN may be involved, we used Ontologizer (8) to determine, for each motif $m$, whether the set of proteins in *core*(${V_m}$) is enriched for particular Gene Ontology terms, i.e. molecular functions, biological processes or cellular components (with the complete set of proteins $V$ as background).

### 5′ UTR motif strand specificity

In order to evaluate the possibility of a motif $m$ to play a functional role at the mRNA level rather than the DNA level (i.e. post-transcriptionally rather than transcriptionally), we measured its strand specificity $ss$, defined as the ratio of the number of occurrences of $m$ to the number of occurrences of its reverse complement in all 5′ UTRs represented in the network. The expectation is that post-transcriptional regulatory motifs have a high strand specificity (>1), whereas most transcriptional regulatory elements, whose function is often independent of strand orientation, may have a strand specificity close to 1. The statistical significance of a strand specificity $ss$ of a motif $m$ is assessed by computing a *P*-value from the cumulative distribution of the normal distribution $\mathcal{N}( {np,\sqrt {np(1 - p)} } )$, which approximates the binomial distribution ${\cal B}(n,p)$, where $n$ is the sum of the number of occurrences of $m$ in the positive and reverse strands and $p\ = \ 0.5$. The *P*-values are then adjusted for multiple hypothesis testing with the Benjamini–Hochberg procedure (49). A motif with a strand specificity adjusted *P*-value $ <$ 0.05 is considered to be likely to have a post-transcriptional involvement. It is important to note that the strand specificity is calculated from the entire set of sequences in the biological network and not solely from the motif occurrences in the core of a motif. It is therefore only used as a measure to hint at a post-transcriptional role of the motif.

### *In vivo* validation of 5′ UTR motifs

Human cDNAs were obtained from the Mammalian Gene Collection. Missing sequences at the 5′ ends of the cDNAs were added by successive rounds of PCR amplification so that the 5′ UTR regions would correspond to NCBI Reference Sequences for SFRS1 (NM_006924.4), SFRS3 (NM_003017.4), RPS15A (NM_001030009.1), RPL21 (NM_000982.3), RPL4 (NM_000968.3) and RPL27 (NM_000988.3). The resulting amplicons corresponding to the full-length 5′ UTR and complete protein-coding region were cloned into p3xFLAG-CMV-14 expression vector (Sigma-Aldrich).

Site-directed mutagenesis was performed so that the NCGCYAUU motifs located in the 5′ UTR of SFRS1 (Chromosome (Chr) 17, position 56 084 602–56 084 609 as annotated by UCSC Genome Browser on Human Feb. 2009 (GRCh37/hg19) Assembly) and SFRS3 (Chr 6: 36 562 139–36 562 146) were mutated correspondingly to the pattern found in Figure 4 and Supplementary Figure S1. The same procedure was done for the YCGYYAUY motifs of RPS15A (Chr 16: 18 801 643–18 801 650) and RPL21 (Chr 13: 27 825 709–27 825 716) and the UUCCUUUY motifs of RPL4 (Chr 15: 66 797 180–66 797 187) and RPL27 (Chr 17: 41 150 453–41 150 460). Positions in the motifs that were found to be conserved across placental mammals (PhastCons elements (48)) were chosen for mutagenesis.

The expression vectors were transfected into HEK 293 cells using Lipofectamine 2000 (Life Technologies) according to the manufacturer’s specifications. The cell line was obtained from ATCC (CRL-1573) and tested for mycoplasma using MycoAlert detection kit (Lonza). The DNA used in these experiments was reduced to 1/20th of the recommended amount in an effort to prevent possible artifactual effects that might stem from over expression of these transcripts. The next day, cells were harvested and lysed with Radioimmunoprecipitation assay (RIPA) buffer (150 mM NaCl; 1% NP-40; 0.5% sodium deoxycholate; 0.1% sodium dodecyl sulphate (SDS); 50 mM Tris, pH 8.0; cOmplete protease inhibitor cocktail (Roche)).

Twenty micrograms of proteins from the cell lysate were separated by SDS-polyacrylamide gel electrophoresis. Following electrotransfer to a PVDF membrane, western blotting was performed using primary anti-FLAG antibody (M2; Sigma-Aldrich: F3165) and anti-β tubulin antibody (TUB 2.1; Sigma; sc-58886) or anti-GAPDH antibody (FL-335; Santa Cruz; sc-25778) as loading controls. Secondary anti-mouse IgG antibody linked to horseradish peroxidase (GE Healthcare; NA931V) was used for detection. The membranes were then incubated with enhanced chemiluminescence (ECL) prime western blotting detection reagent (GE Healthcare) and scanned using an ImageQUANT LAS-4000 biomolecular imager (GE Healthcare). Relative fluorescence units corresponding to the amounts of expressed FLAG-tagged proteins were determined with ImageQuant TL 1-D gel analysis tool (Version 8.1). The statistical significance assessment of the differential expression between mutants and wild-type (WT) was performed using a two-tailed unpaired Student’s *t*-test.

### Implementation and availability

The proposed computational tools are implemented in a platform independent Java program called LESMoN. LESMoN along with the complete GO enrichment analysis results for the 1873 motifs with clustering *P*-values < 10^−6^ and the nine motifs identified with the alternative method are available as supporting material for download at: http://www.cs.mcgill.ca/∼blanchem/LESMoN.

## RESULTS

**Figure 2.**
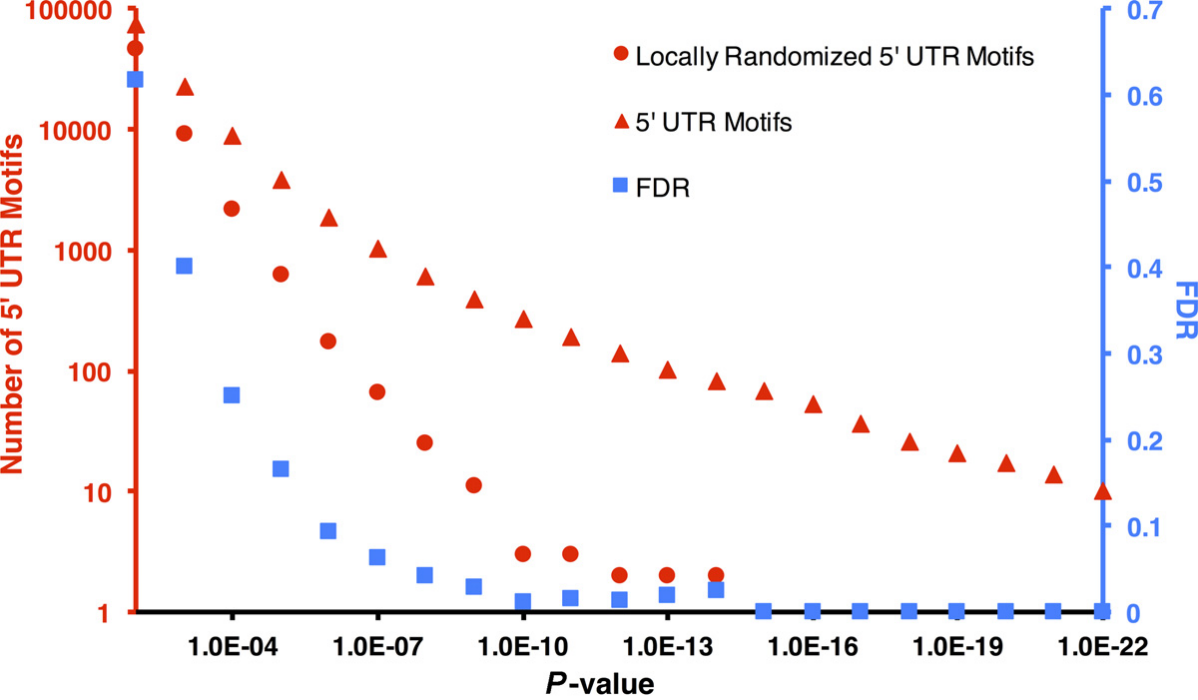
Number of motifs originating from both actual and locally randomized 5′ UTR sequences (circular and triangular markers) and false discovery rate (FDR) for a given clustering *P*-value threshold (square markers, on the secondary axis) for top percent pairwise distance (TPPD) of top 10%.

LESMoN is an approach that identifies short sequence motifs that occur in a set of sequences that are clustered with respect to a given biological network. Specifically, LESMoN takes as input an undirected biological network $G\ = \ ( {V,E} )$, where each node $v$$ \in$$V$ is associated with a sequence. In this paper, LESMoN is applied to the BioGRID protein–protein interaction network (37,38) and the sequences associated with proteins are the 5′ UTRs. The network contains 12 133 proteins and 94 490 unique pairwise interactions identified using various technologies and experimental protocols (see ‘Materials and Methods’ section). A set of 3 558 817 mRNA motifs of length 8 were evaluated for clustering in $G$. Figure 2 shows the number of motifs identified at various clustering *P*-value thresholds using the top 10% TPPD ($l = 0.1 \cdot |{V_m}|$; see ‘Materials and Methods’ section). 1873 motifs obtained a *P*-value < 10^−6^, which corresponds to a FDR <10% (estimated based on locally permuted 5′ UTR sequences, see ‘Materials and Methods’ section). We selected this set of motifs for further analyses (See Supplementary Table S1 for a list of all identified motifs). We also note that 269 motifs obtained a *P*-value below 10^−10^, which corresponds to a lower FDR (<0.02) (See Supplementary Methods and Supplementary Figure S2 for more details on the clustering *P*-value distribution). We also evaluated the performances of LESMON using the top 5% (resp. 20%) TPPD and found inferior prediction power, obtaining only 1211 (resp. 528) significant motifs (FDR <10%; see ‘Materials and Methods’ section and Supplementary Figures S3–5), 88% of which were also identified using the top 10% TPPD. Indeed, top 5% TPPD appears to not be as discriminative as top 10% for the differentiation of the different levels of clustering in the network. On the other hand, top 20% seems to capture too much noise in the clustering measure for a given motif.

**Figure 3.**
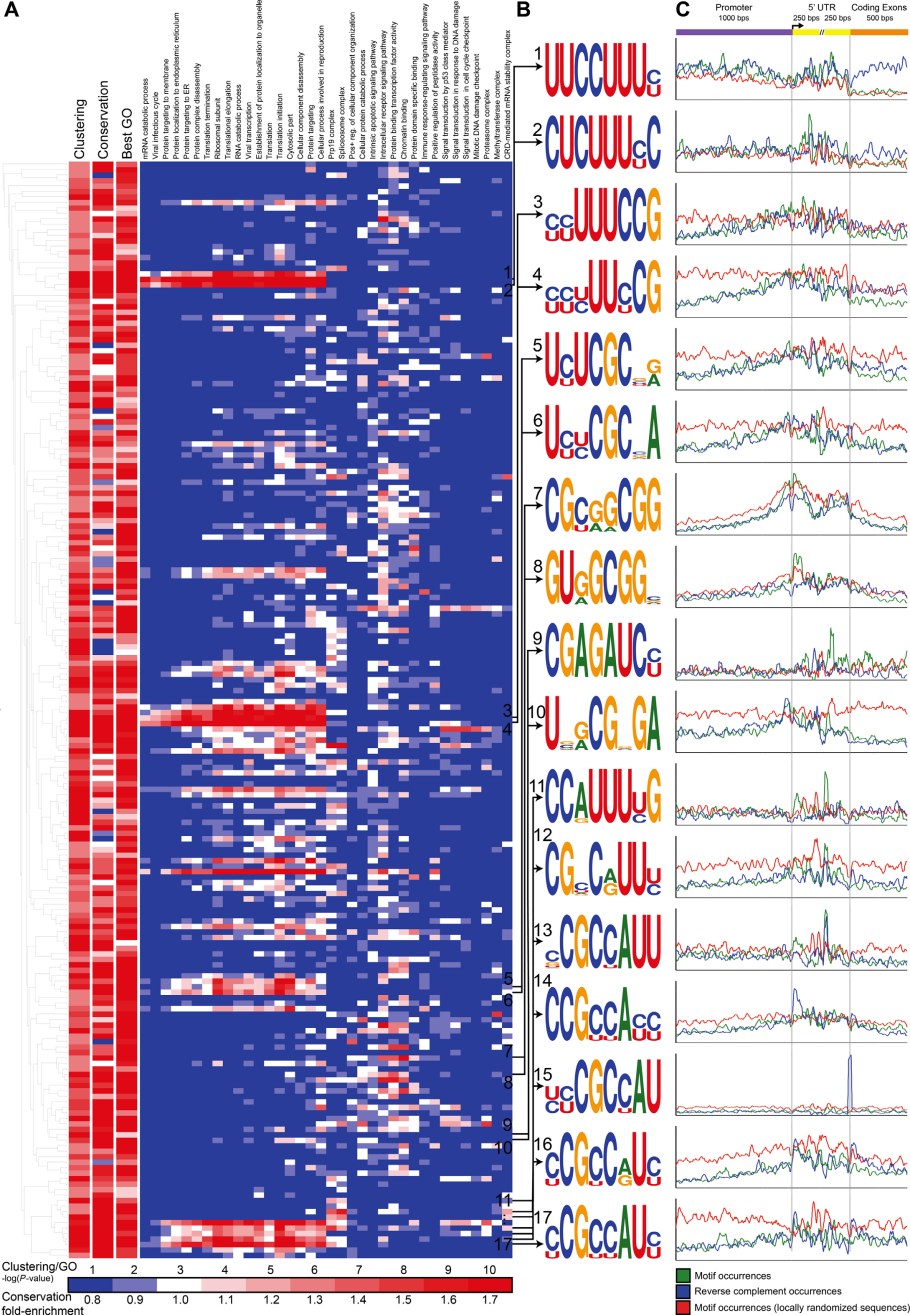
Significantly clustered 5′ UTR motifs in the BioGRID human protein–protein interaction network. (**A**) LESMoN identified 200 motif family representatives with clustering *P*-values < 10^−6^ that are displayed in a hierarchical clustering tree. Conservation fold enrichment, clustering and GO enrichment *P*-values for each motif are color-coded. GO enrichment *P*-values were computed with Ontologizer (8) using a Fisher’s exact test. The 36 GO terms shown here are those that are significantly (*P*-value < 10^−7^) associated with the most motifs, considering only terms that include $ \le$ 500 human genes. (**B**) The family representative motifs with a conservation fold enrichment $ \ge$ 2.25 are shown as sequence logos (generated by Weblogo (44)), where nucleotide heights are proportional to their frequencies in 5′ UTRs. Each represented motif is given an identification number (from 1 to 17). (**C**) For these 17 motifs, the motif and its reverse complement occurrences in promoters, 5′ UTRs and coding exons in actual and locally randomized sequences are shown.

If a sequence motif $m$ is deemed significantly clustered in the network by LESMoN, a more specific version of $m$ or a motif similar to $m$ is likely to also be found significant. To reduce the redundancy in the set of 1873 motifs identified by LESMoN, we used a hierarchical clustering algorithm based on the similarity of the sets of proteins for which the 5′ UTR sequences contain the motifs (See ‘Materials and Methods’ section). This resulted in the identification of 200 motif families, ranging in size from 1 to 149 motifs (Figure 3A). For each family, the motif with the lowest clustering *P*-value was retained as the representative motif (Supplementary Table S2). Finally, for each of these motifs we defined the core of a motif as the subset of proteins associated to the motif that are the most clustered within the network (see ‘Materials and Methods’ section).

### LESMoN identifies evolutionarily conserved 5′ UTR motifs

Interspecies sequence conservation is generally evidence of function (50–52) and functional portions of 5′ UTRs have been mapped based on this principle (53–55). To assess the biological relevance of each of the motifs identified by LESMoN, we determined the fraction of matching sequences that overlaps regions that are highly conserved within placental mammals (PhastCons elements (48)) and computed a conservation fold-enrichment by comparing it to the overall fraction of 5′ UTR nucleotides that are highly conserved (27%; see ‘Materials and Methods’ section). The motif occurrences in the cores of >54% of the 200 motif family representatives had a high conservation fold-enrichment (>1.5) (Figure 3A). This suggests that occurrences of motifs that are clustered in the network are often evolutionary conserved and therefore likely to be biologically functional. Figure 3B presents the 17 motifs for which the conservation fold-enrichment was ≥2.25. This high fold-enrichment threshold was selected for presentation purposes to narrow down the list of motifs to those with strongest evidence of selection. These motifs will be analyzed in greater depth below. The remaining significantly clustered motifs are reported in Supplementary Table S2.

### Positional enrichment and strand specificity of 5′ UTR motifs

Even though motifs found by LESMoN are present in 5′ UTRs, their primary function may still be as transcriptional regulators at the DNA level. We posit that motifs whose density is higher in 5′ UTRs than in flanking promoters (See Figure 3C) are more likely to be involved in post-transcriptional regulation. Figure 3C provides the occurrence profiles of the 17 selected motifs, as well as that of their reverse complement, in promoters, 5′ UTRs, coding exon sequences and locally randomized sequences. Upon visual inspection of these occurrence profiles, we notice that the motifs are sometimes enriched toward the beginning or toward the end of the 5′ UTRs. For some motifs, such as GURGCGGN (motif 8, Figure 3B) and NCGCYAUU (motif 13, Figure 3B), the occurrences suddenly increase immediately downstream of the TSS, suggesting a post-transcriptional role, while other motifs, such as CGYRRCGG (motif 7, Figure 3B) and UNRCGNGA (motif 10, Figure 3B) are more symmetrically distributed around the TSS, suggesting a role in transcriptional regulation. Table 1 lists these 17 motifs along with supplementary information such as the strand specificity (See ‘Materials and Methods’ section) and curated GO term enrichments of their associated core proteins.

**Table 1.** Family representative motifs identified by LESMoN with a conservation fold enrichment $ \ge$ 2.25

Motif	Number of proteins	Clustering *P*-value	Conservation fold enrichment	Strand specificity	Strand specificity adjusted *P*-value	Curated GO enrichments
CCRUUUYG	172	1.15 $ \times$ 10^−12^	3.27	2.29	6.95 $ \times$ 10^−9^	RNA processing: 5.0 $ \times$ 10^−8^
						Prp19 complex: 6.3 $ \times$ 10^−6^
CGAGAUCY	73	8.40 $ \times$ 10^−17^	3.24	1.03	0.690	Transcription factor binding: 9.4 $ \times$ 10^−5^
NCGCYAUU	227	7.31 $ \times$ 10^−17^	3.22	1.68	6.96 E $ \times$ 10^−6^	Ribonucleoprotein complex: 1.9 $ \times$ 10^−9^
						Spliceosomal
						complex: 1.5 $ \times$ 10^−7^
YCGYYAUY	424	1.35 $ \times$ 10^−30^	2.72	1.25	0.004	Translational
						initiation: 7.3 $ \times$ 10^−17^
						Translational
						Elongation: 2.8 $ \times$ 10^−14^
						Ribosome: 1.7 $ \times$ 10^−12^
YYCGCYAU	220	2.23 $ \times$ 10^−16^	2.70	1.76	1.37 $ \times$ 10^−6^	Translational
						initiation: 3.1 $ \times$ 10^−9^
						Ribosomal
						subunit: 8.1 $ \times$ 10^−8^
UUCCUUUY	270	3.42 $ \times$ 10^−35^	2.68	0.94	>0.999	Ribosomal
						subunit: 8.8 $ \times$ 10^−31^
						Viral
						transcription: 2.6 $ \times$ 10^−24^
						RNA catabolic
						process: 3.8 $ \times$ 10^−23^
						Protein targeting to ER: 2.8 $ \times$ 10^−18^
CUCUUUYC	269	2.01 $ \times$ 10^−17^	2.61	1.07	0.430	Translational
						initiation: 3.0 $ \times$ 10^−23^
						Ribosomal
						subunit: 5.7 $ \times$ 10^−20^
YYUUUCCG	252	3.71 $ \times$ 10^−19^	2.47	1.31	0.008	Translational
						initiation: 4.1 $ \times$ 10^−23^
						Ribosomal
						subunit: 3.1 $ \times$ 10^−17^
YYYUUYCG	785	5.85 $ \times$ 10^−15^	2.46	1.09	0.113	Translational
						initiation: 6.3 $ \times$ 10^−30^
						Ribosomal
						subunit: 1.1 $ \times$ 10^−27^
						Viral
						transcription: 2.3 $ \times$ 10^−24^
YCGYCRUY	794	6.35 $ \times$ 10^−12^	2.41	1.13	0.029	mRNA metabolic
						process 6.4 $ \times$ 10^−13^
UNRCGNGA	868	2.13 $ \times$ 10^−9^	2.41	1.13	0.016	Nucleus: 2.6 $ \times$ 10^−10^
						Cell cycle: 1.0 $ \times$ 10^−8^
UYYCGCNA	491	1.38 $ \times$ 10^−7^	2.41	0.86	>0.999	Translational
						initiation: 5.3 $ \times$ 10^−11^
						Ribosomal
						subunit: 4.0 $ \times$ 10^−10^
GURGCGGN	980	3.09 $ \times$ 10^−13^	2.33	1.38	7.05 $ \times$ 10^−11^	Nucleus: 2.4 $ \times$ 10^−10^
						Chromosome
						organization: 4.1 $ \times$ 10^−6^
						Transcription from RNA polymerase II
						promoter: 4.1 $ \times$ 10^−6^
UYUCGCNR	610	7.74 $ \times$ 10^−10^	2.30	1.09	0.150	Translation
						initiation: 3.6 $ \times$ 10^−10^
						Reproduction: 1.8 $ \times$ 10^−7^
CGNCRUUY	458	2.68 $ \times$ 10^−7^	2.29	1.44	2.83 $ \times$ 10^−6^	mRNA
						processing: 4.9 $ \times$ 10^−13^
						Spliceosomal
						complex: 6.9 $ \times$ 10^−10^
						CRD-mediated mRNA stability complex: 1.8 $ \times$ 10^−6^
CGYRRCGG	1196	9.12 $ \times$ 10^−7^	2.27	1.12	0.009	Transcription factor binding: 5.4 $ \times$ 10^−11^
						Chromatin
						binding: 5.6 $ \times$ 10^−11^
						Death: 2.7 $ \times$ 10^−9^
CCGYYAYY	963	1.43 $ \times$ 10^−11^	2.25	0.88	>0.999	mRNA metabolic
						process: 2.7 $ \times$ 10^−15^

### Proteins associated with evolutionary conserved motifs identified by LESMoN are significantly enriched with multiple GO terms

To further investigate the biological significance of each motif $m$ identified by LESMoN, we asked if GO terms were enriched in the set of proteins of the core associated with $m$. Figure 3A shows a subset of the GO terms that were found to be enriched in the set of proteins associated with the 200 family representative motifs (See ‘Implementation and Availability’ section). A total of 72% of the motif family representatives, including all of the 17 selected motifs, are associated with proteins enriched for at least one GO term (Corrected enrichment *P*-value < 0.001; Figure 3A). Table 1 reports curated GO terms for these 17 conserved motifs. As mentioned previously, 5′ TOP motifs, which are CU-rich motifs located at the 5′ end of 5′ UTRs, are known to regulate mRNAs of proteins involved in translation and elongation. Motifs matching or highly resembling 5′ TOP motifs were found to be significantly clustered by LESMoN, since the proteins they are associated with, mostly ribosomal proteins, are tightly interacting in the protein–protein interaction network (32). Four of the highly conserved family representative motifs, namely UUCCUUUY (motif 1, Figure 3B), CUCUUUYC (motif 2, Figure 3B), YYUUUCCG (motif 3, Figure 3B) and YYYUUYCG (motif 4, Figure 3B) are associated with proteins that are statistically significantly enriched for GO terms related to the ribosome and translation. Of note, all of these motifs are associated with a strand specificity close to 1, with the exception of YYUUUCCG (motif 3, Figure 3B; strand specificity = 1.31, adjusted *P*-value = 0.008) hinting that they might be involved in transcriptional regulation, a behavior that is not typically associated to 5′ TOP motifs. The YYUUUCCG motif does however contain the 5′ TOP element of RPS27 (5′-**CUUUCCG**). Interestingly, it was previously reported that the mutation from a C to a U at Chr1: 153963239, the TSS of RPS27, causes the 5′ TOP motif to expand (5′-**CUUUUCCG**) and is associated with a higher frequency of melanomas (56).

Five of the seventeen highly conserved motifs (NCGCYAUU (motif 13, Figure 3B), CCRUUUYG (motif 11), CGNCRUUY (motif 12), CCGYYAYY (motif 14) and YCGYCRUY (motif 16) were found to be significantly enriched with GO terms related to mRNA processing and splicing, suggesting that such motifs may be involved in the regulation of the spliceosomal machinery. NCGCYAUU (motif 13, strand specificity = 1.68, adjusted *P*-value = 6.96⋅10^–6^), CCRUUUYG (motif 11, strand specificity = 2.29, adjusted *P*-value = 6.95⋅10^–9^) and CGNCRUUY (motif 12, strand specificity = 1.44, adjusted *P*-value = 2.83⋅10^–6^) are all associated with high strand specificity, suggesting a post-transcriptional role for these motifs.

In addition, proteins associated with the closely related YCGYYAUY (motif 17, strand specificity = 1.25, adjusted *P*-value = 0.004), YYCGCYAU (motif 15, strand specificity = 1.76, adjusted *P*-value = 1.37⋅10^–6^), UYYCGCNA (motif 6, strand specificity = 0.86, adjusted *P*-value $ \simeq$ 1) and UYUCGCNR (motif 5, strand specificity = 1.09, adjusted *P*-value = 0.15) motifs were found to be enriched with ribosomal proteins and translation related GO terms. While these motifs are CU-rich, they do not share the profile of typical 5′ TOP motifs. This finding suggests an alternative regulatory motif for some ribosomal proteins. Both YCGYYAUY (motif 17, Figure 3B) and YYCGCYAU (motif 15, Figure 3B) presented high strand specificities (Table 1). Other motifs such as GURGCGGN (motif 8, Figure 3B) and UNRCGNGA (motif 10, Figure 3B) showed a significant enrichment for proteins localized in the nucleus. In addition, GURGCGGN (motif 8, Figure 3B) is associated with a high strand specificity (1.38, adjusted *P*-value = 7.05⋅10^–11^). These results could hint at a potential mRNA localization role of such a motif in 5′UTRs, even though the majority of known perinuclear localization motifs are situated in 3′UTRs (57). Defining the precise role of this motif would require additional experiments and is beyond the scope of this study.

### Benchmark against GoNet and conventional motif discovery method

We attempted to identify clustered 5′UTR motifs in the human BioGRID PPI network using our previously published approach GoNet. To this end, GO terms were replaced by the set of 5′ UTR motifs of length 8. As discussed in the ‘Materials and Methods’ section, GoNet methodological limitations prevented the detection of 5′ UTR motifs that are clustered in the network. We also benchmarked our approach on the human BioGRID PPI network by submitting it to the MCL and using the MEME software package (see ‘Materials and Methods’ section) to identify enriched motifs in each of the clusters identified. MCL identified 1810 clusters containing at least 2 proteins, from which MEME identified nine 5′ UTR motifs with a FDR = 22% (Supplementary Table S3 and Figure S6). LESMoN clearly displays a greater sensitivity and identified more 5′ UTR motifs than this conventional motif discovery strategy. This is likely due to the fact that LESMoN has the ability to analyze in their entirety large overlapping clusters that are broken into smaller clusters by the MCL analysis. Interestingly, the two motifs that obtained the lowest *E*-values with the MEME analysis ([UC]UC[UC]UU[UC][CU] and UUU[UAG][CUG][UA]UU) correspond to motifs that were also detected by LESMoN: CUCUUUYC and UUCCUUUY (Table 1) for the first MEME motif and YUUYCUUU (Supplementary Table S1) for the second. Of all nine motifs identified with the alternative approach (MEME *E*-value < 1), only UUCC[GU]G[UC][GC] had a conservation fold-enrichment >1.5. However, all motifs showed an enrichment (Corrected enrichment *P*-value < 0.001) for at least one GO term (Supplementary Figure S6A and see ‘Implementation and Availability’ section for complete results). The motif with the lowest *E*-value was very significantly enriched for a large number of GO terms involved in translation and protein localization. Finally, those motifs are generally somewhat uniformly distributed across the 5′ UTR sequences, with the exception of UUCC[UG]G[CU][CG] that appears to be enriched toward the 5′ end of 5′ UTRs (Supplementary Figure S6C). Five of those 5′ UTR motifs, UUU[UAG][CUG][UA]UU, [AG]A[AG]GAA[AG]A, UUCC[GU]G[UC][GC], AGAGA[AU]GA and U[CU]AU[CU]UUU have a high strand specificity (strand specificity of 1.26, 1.23, 1.24, 1.39, 1.35 and a *P*-value of 1.39⋅10^–9^, 3.6⋅10^–8^, 9.52⋅10^–6^, 3.75⋅10^–4^, 7.46⋅10^–4^, respectively), hinting at their potential role at the transcript level.

### *In vivo* validation of the biological role of 5′UTR motifs discovered by LESMoN

**Figure 4.**
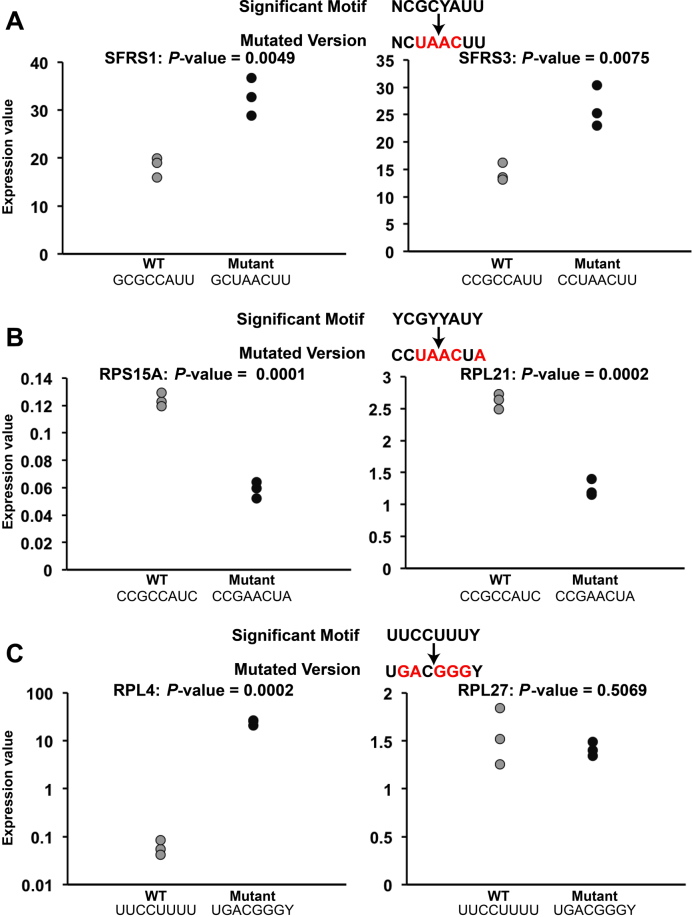
Western blot analysis in HEK 293 cells of the expression of proteins with their associated wild-type (WT) 5′ UTRs and mutated 5′ UTRs at positions discovered by LESMoN. The *P-*values were calculated using an unpaired two-tailed Student’s *t*-test. (**A**) SFRS1 and SFRS3. (**B**) RPS15A and RPL21. (**C**) RPL4 and RPL27.

We experimentally assessed the biological function of 3 of the 17 conserved family representative motifs identified by LESMoN (NCGCYAUU (motif 13), YCGYYAUY (motif 17) and UUCCUUUY (motif 1). These motifs were selected based on their highly significant clustering and GO enrichment *P*-values, on their great conservation fold enrichments and on the availability of constructs for their associated genes. For each motif, we mutated two of their occurrences (three biological replicates of different cell cultures for each condition: mutated and WT). The NCGCYAUU motif (motif 13, Figure 3B) appears in the 5′ UTRs of two serine/arginine-rich splicing factor, SFRS1 (motif: GCGCCAUU) and SRFS3 (motif: CCGCCAUU), which encode proteins that are part of the splicing machinery. Upon mutation of these motif occurrences, the expression of both splicing factors was significantly increased (two-tailed unpaired Student’s *t*-test *P*-values of 0.0046 and 0.0075, respectively), suggesting a repression role of the NCGCYAUU motif (Figure 4A and Supplementary Figure S1A). Of note, this change in protein expression might not be directly linked to translation regulation, but can also be caused by a change in localization or stability of the mRNAs. These findings are particularly interesting as it provides insights about the regulation mechanisms of the mammalian splicing machinery. We mutated the YCGYYAUY motif (motif 17, Figure 3B) in Ribosomal Protein S15a (RPS15A; motif: CCGCCAUC) and in Ribosomal Protein L21 (RPL21; motif: CCGCCAUC) and observed a significant decrease in expression for both ribosomal proteins (*P*-value = 0.0001 and 0.0002, respectively) (Figure 4B and Supplementary Figure S1B). The YCGYYAUY motif may therefore be involved in the positive regulation of ribosomal proteins and of the translation machinery. Finally, we mutated the UUCCUUUY motif (motif 1, Figure 3B), which closely resembles 5′ TOP motifs. We mutated UUCCUUUU in Ribosomal Protein L4 (RPL4), where the motif starts 7 bases away from the 5′ end of the UTR. This location is close to but not the typical location of a 5′ TOP motif, which occurs exactly at the 5′ end of UTRs. The RPL4 mutant showed a statistically significant increase of expression (*P*-value = 0.0002) (Figure 4C and Supplementary Figure S1C). This result is in agreement with the translation inhibition role of 5′TOP motifs that was previously described in the literature (58–60). We also mutated the motif in Ribosomal Protein L27 (RPL27). The motif occurs close to the 5′ end of the UTR and appears to be part of a large 5′ TOP motif (5′-UCCUUCU**UUCCUUUU**U). However, no significant changes were observed for the mutant of RPL27 (Figure 4C and Supplementary Figure S1C). This may be caused by the larger length of the motif, such that the first half of the motif (which was not mutated) may compensate for the loss of the second half.

## DISCUSSION

In this paper, we propose an approach to identify 5′ UTR sequence motifs for which the associated proteins are significantly clustered in a given PPI network. We also presented a set of computational tools to evaluate the biological relevance of the 5′ UTR motifs identified and validated a number of them *in vivo*. Our approach discovered several previously uncharacterized 5′ UTR motifs and associated them with biological processes taking place in PPI networks. This paper explores one of the many applications of LESMoN. Besides 5′ UTRs, 3′ UTRs could also be analyzed in the same fashion to potentially identify novel mRNA localization signals. In addition, LESMoN could analyze different types of sequences, such as introns, coding exons or promoter sequences. The latter could be interesting especially for the discovery of transcription factor binding sites regulating the transcription of proteins interacting in the cell. Amino acid sequences could also be considered for the discovery of peptide sequences mediating protein localization or protein–protein interactions (See Supplementary Discussion for more alternative methodological approaches that could be used for LESMoN).

The connectivity of the biological network used as input by LESMoN impacts its ability to identify clustered 5′ UTR motifs. In densely connected networks, LESMoN is less likely to identify clustered motifs because all proteins are heavily connected. In the contexts of PPIs, it is important to note that network connectivity does not necessarily correlates with quality. The varying level of connectivity in PPI networks can be explained by the type of experimental methods used to obtain the PPIs. Methods such as yeast-two-hybrid screening (61,62) and tandem affinity purification (TAP) coupled to mass spectrometry (63–65) tend to produce more direct interactions than the BioID (66,67) technique and the FLAG affinity purification coupled to mass spectrometry (68). A large fraction of the BioGRID database is composed of PPIs obtained from yeast-two-hybrid and TAP. On the opposite, STRING includes many indirect and computationally predicted PPIs. This explains in part the contrast between the very dense STRING network (69) versus the sparser BioGRID network. Whereas LESMoN identified a large number of statistically significant and biologically relevant motifs based on the BioGRID network, it did not detect many significantly motifs based on the STRING network (data not shown). This suggests that in order to take advantage of very dense networks, the TPPD percentage may need to be determined based on the distribution of the protein degrees in the network, but more likely, alternative measures of clustering should be considered, e.g. those based on Markov random walks (9) or those taking advantage of confidence values assigned to edges of the network.

RNA molecules are known to form various secondary structures in order to perform their functions, which often consist in binding proteins or other RNAs. In this article, we opted to only consider the primary structure of 5′ UTRs, but our approach could be extended to study RNA secondary structure motifs, such as a 6 nt hairpin loop or a bulge of 2 nt. This approach could be beneficial since RNA sequences may differ but still form similar RNA secondary structures, such as the 3′ UTR teloplasm localization motif, which is necessary for the proper localization of *Hro-twist* mRNA in leech (70).

Our method could also be extended to perform protein function prediction. Often, several proteins among those found by LESMoN to be clustered and associated with the same 5′ UTR motif are uncharacterized. LESMoN provides crucial pieces of information to infer the function of these uncharacterized proteins and brings an additional dimension to the ‘guilt by association’ approach for protein function prediction (71–74). A strategy could be implemented to compute likelihoods for such uncharacterized proteins to perform a certain function based on their co-clusterings with already functionally annotated proteins and the shared occurrence of a given 5′ UTR motif.

This paper is a first step in the general direction of using networks to identify functional sequence features through local enrichment. Networks can capture a variety of biological relationships in a much richer manner than gene sets or ranked gene lists can. As such, using them to identify functional motifs should prove particularly fruitful, as our results on 5′ UTR motifs suggest. While we focused in this paper on the analysis of PPI networks, metabolic or regulatory networks could provide equally interesting insights. In addition, computationally created correlation networks (co-expression, co-methylation, etc.) may also be mined for regulatory motifs, which may yield deeper insights into their complex structure and the molecular mechanisms driving them.

## DATA AVAILABILITY

The proposed computational tools are implemented in a platform-independent Java program called LESMoN. LESMoN along with the complete GO enrichment analysis results for the 1873 motifs with clustering *P*-values < 10–6 and the nine motifs identified with the alternative method are available as supporting material for download at: http://www.cs.mcgill.ca/∼blanchem/LESMoN.

## Supplementary Material

Supplementary Data
