# Supplementary material for: Functional 5′ UTR motif discovery with LESMoN: Local Enrichment of Sequence Motifs in biological Networks
Source: Nucleic Acids Res. 2017 Aug 31;45(18):10415–27. doi: 10.1093/nar/gkx751 (PMC5737372; doi:10.1093/nar/gkx751)
Supplement: Supplementary Data [file gkx751_supp.zip › nar-01137-n-2016-File006.pdf]

Functional 5' UTR Motif Discovery with LESMoN: Local Enrichment of Sequence Motifs in Biological Networks

Mathieu Lavallée-Adam[1,2], Philippe Cloutier[3], Benoit Coulombe[3,4] and Mathieu Blanchette[1,*]

[1] McGill Centre for Bioinformatics and School of Computer Science, McGill University, Montréal, Québec, H3A 2B2, Canada
[2] Ottawa Institute of Systems Biology and Department of Biochemistry, Microbiology and Immunology, Faculty of Medicine, University of Ottawa, Ottawa, Ontario, K1H 8M5, Canada,.
[3] Translational Proteomics Laboratory, Institut de recherches cliniques de Montréal, Montréal, Québec, H2W 1R7, Canada
[4] Département de biochimie et médecine moléculaire, Université de Montréal, Montréal, Québec, H3C 3J7, Canada

* To whom correspondence should be addressed. Tel: 1-514-398-5209; Fax: 1-514-398-3883; Email: blanchem@cs.mcgill.ca

SUPPLEMENTARY METHODS

Clustering p-value distribution

Although this does not affect the accuracy of the FDR estimates, we note that the distribution of p-values obtained in the locally randomized sequences is not quite uniform (Supplementary Figure S2). For example, in these locally randomized sequences, we found 173 motifs with p-values below 10^{-6}, whereas only ~4 would have been expected to occur by chance. This appears to be due to differences in sequence compositions (most likely GC content) between the 5' UTRs of the genes encoding the proteins in different portions of the network. Indeed, when the procedure is repeated on 5' UTR sequences that are completely randomized (equal occurrence probability of each nucleotide), the distribution of p-values is very close to uniform (Supplementary Figure S2).

SUPPLEMENTARY DISCUSSION

Alternative methodological approach using LESMoN

While LESMoN is sensitive enough to detect numerous 5' UTR sequence motifs of potential biological interest, it could be improved to reach an even higher sensitivity. In the present state, the approach only explores motifs constituted of at most 8 characters. With such length, random occurrences of motifs in 5' UTRs are fairly frequent, making the set of proteins associated with a motif noisy. By increasing motif length, longer 5' UTR motifs with a biological role may emerge from this noise. Longer motifs will be associated with a smaller set of proteins that are clustered with a greater significance in the network. Expanded sets of degenerate characters (e.g. K=G|T), M=A|C, S=G|C, and W=A|T), may also be considered. However, in both cases, the exponential growth of the number of motifs as a function of their length or alphabet size translates into difficulties on both the computational (running time) and statistical (multiple hypothesis testing)

aspects. This could be alleviated by incremental motif discovery, where short motifs are identified first and then significant ones are considered for extension to larger lengths. We could also consider different sampling strategies in order to build the null distributions of the TPPD. Methods sampling k random proteins sharing in their sequences a similar nucleic acid distribution or a motif similar to the one being evaluated could provide a more realistic null distribution of the TPPD. Finally, the identification of the motif families could also be improved. The cut in the tree could be performed using a bootstrapping approach assessing the significance of motif clusters that could potentially unravel motifs that are currently missed by LESMoN.

SUPPLEMENTARY FIGURES

Supplementary Figure S1. Western blot analysis in HEK 293 cells of the expression of proteins with their associated wild type 5' UTRs and mutated 5' UTRs at positions discovered by LESMoN. Tubulin and GAPDH were used as controls.

A SFRS1 and SFRS3

B RPS15A and RPL21

C RPL4 and RPL27

Supplementary Figure S2. Cumulative distributions of clustering p-values computed by LESMoN using the TPPD of $l = 0.1 \cdot |V_m|$ (Top 10%) for motifs obtained from completely randomized, locally randomized, and unmodified 5' UTR sequences compared to a theoretical uniform distribution.

Supplementary Figure S3. Number of motifs originating from both actual and locally randomized 5' UTR sequences (in red) and FDR for a given clustering p-value threshold (in blue, on the secondary axis) for TPPD of top 5%.

Supplementary Figure S4. Number of motifs originating from both actual and locally randomized 5' UTR sequences (in red) and FDR for a given clustering p-value threshold (in blue, on the secondary axis) for TPPD of top 20%.

Supplementary Figure S5. Number of 5' UTR motifs identified at a given FDR threshold for the top 5%, top 10%, and top 20% TPPD.

Supplementary Figure S6. 5' UTR sequence motifs identified by MEME in MCL clusters from the BioGRID human protein-protein interaction network.

(A) MEME identified 9 motifs in MCL clusters with E-values < 1. E-values, conservation fold enrichment, and GO enrichment p-values for each motif are color-coded. GO enrichment p-values were computed with Ontologizer using a Fisher's exact test. The 18 GO terms shown here are those that are significantly (p-value $<10^{-7}$) associated with the most motifs, considering only terms that include $\leq$ 500 human genes.

(B) Motifs are shown as sequence logos (generated by Weblogo), where nucleotide heights are proportional to their frequencies in 5' UTRs.

(C) The motif and its reverse complement occurrences in promoters, 5' UTRs, and coding exons in actual and locally randomized sequences are shown.

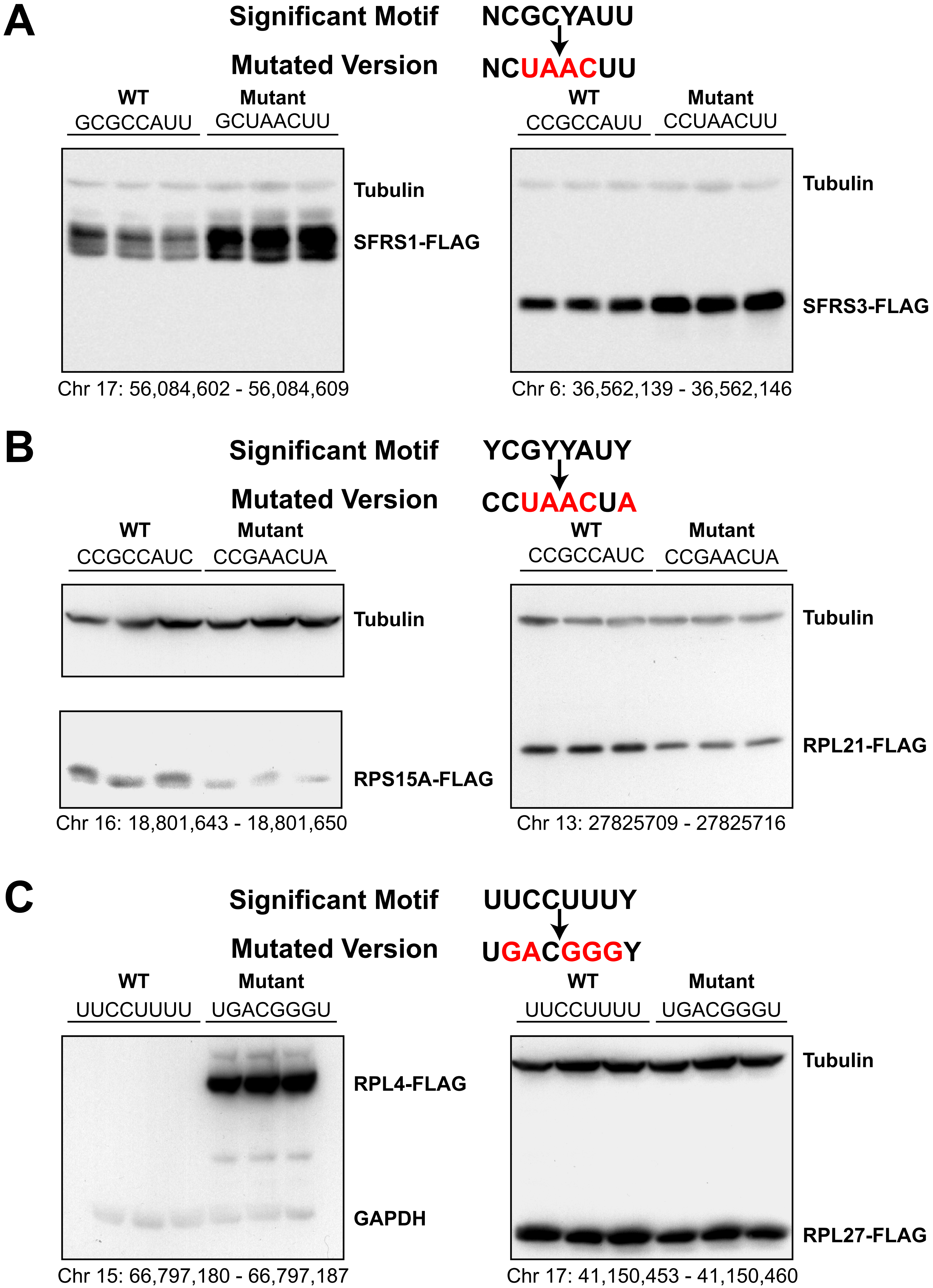

Supplementary Figure S1

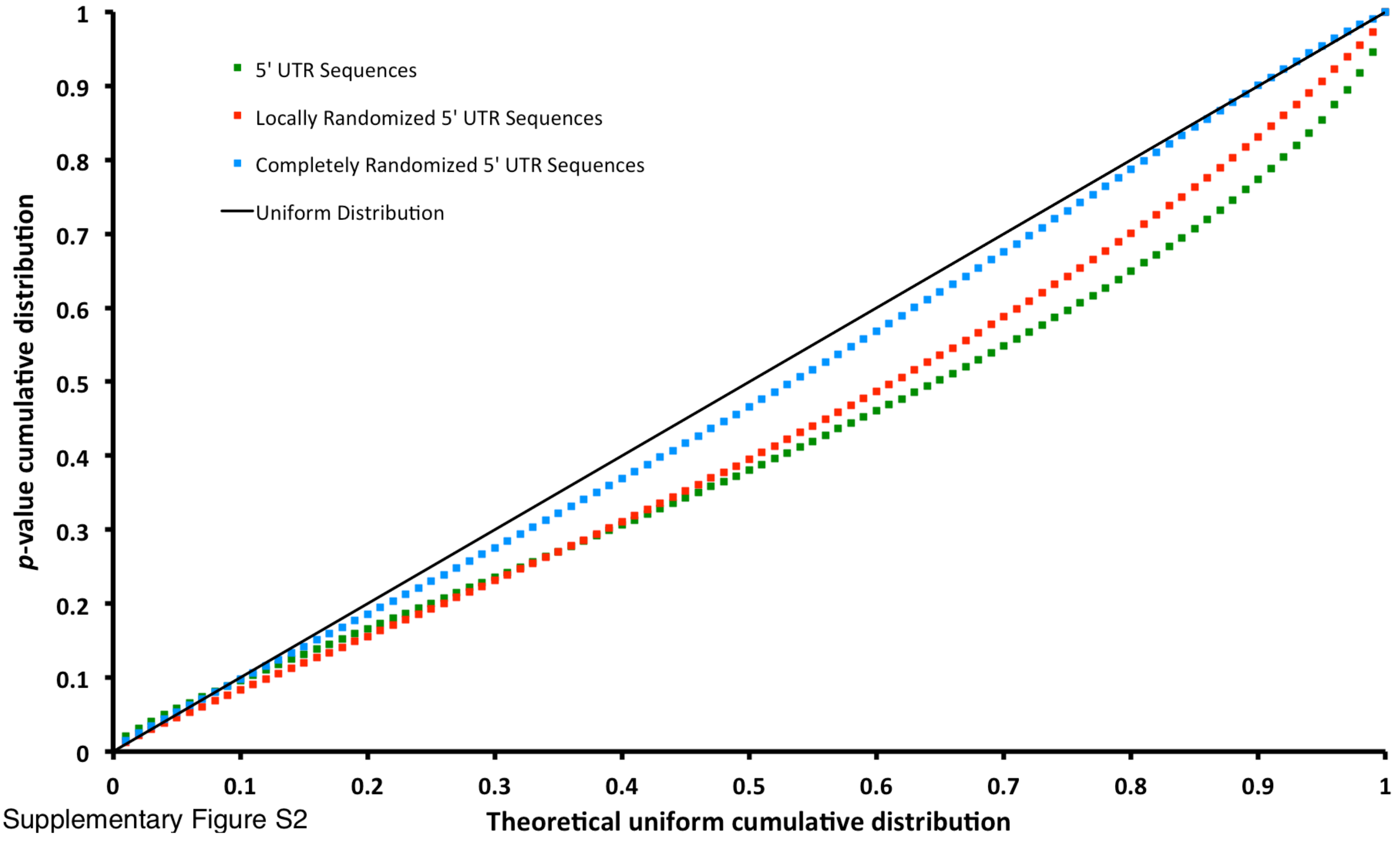

Supplementary Figure S2

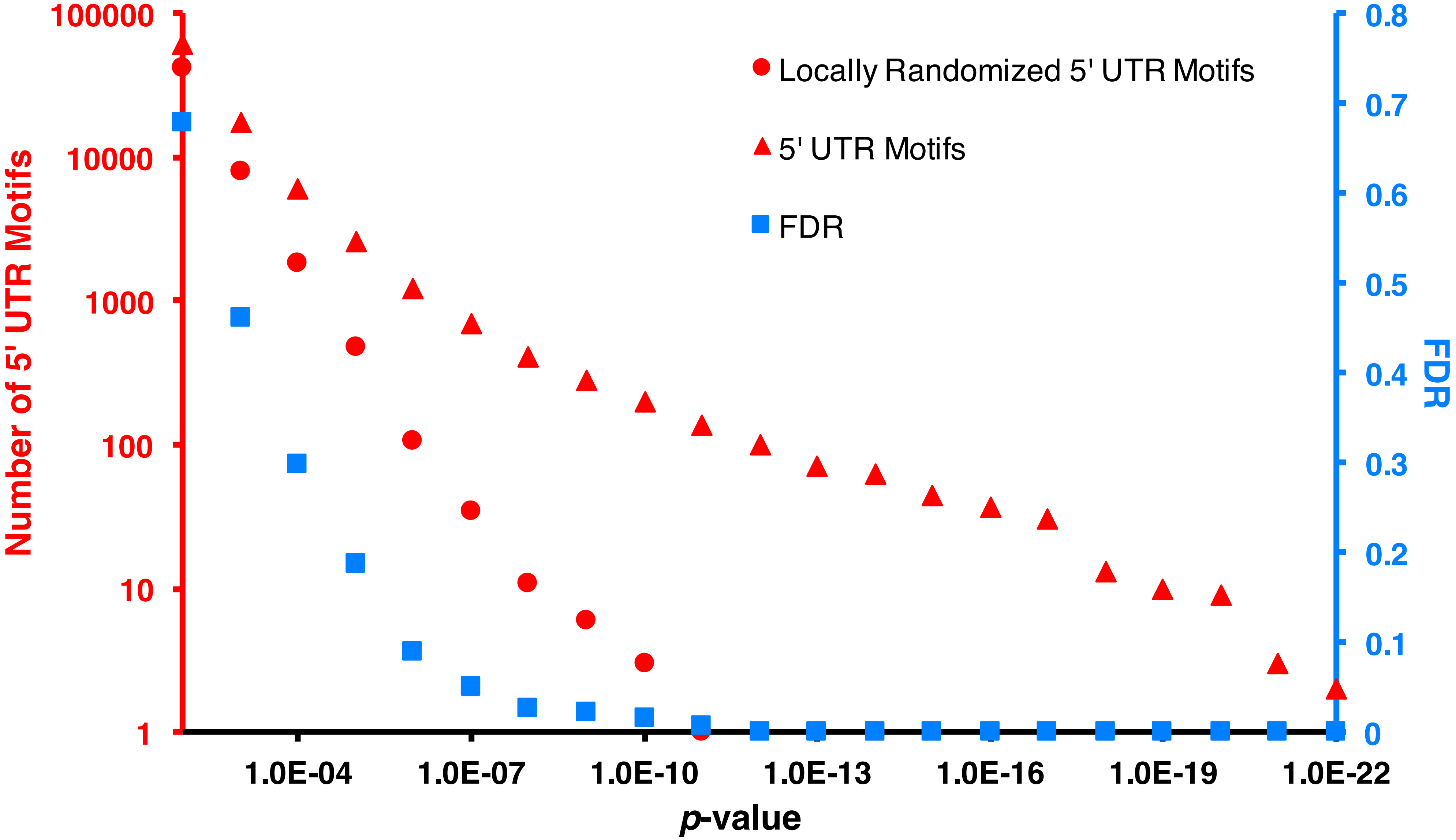

Supplementary Figure S3

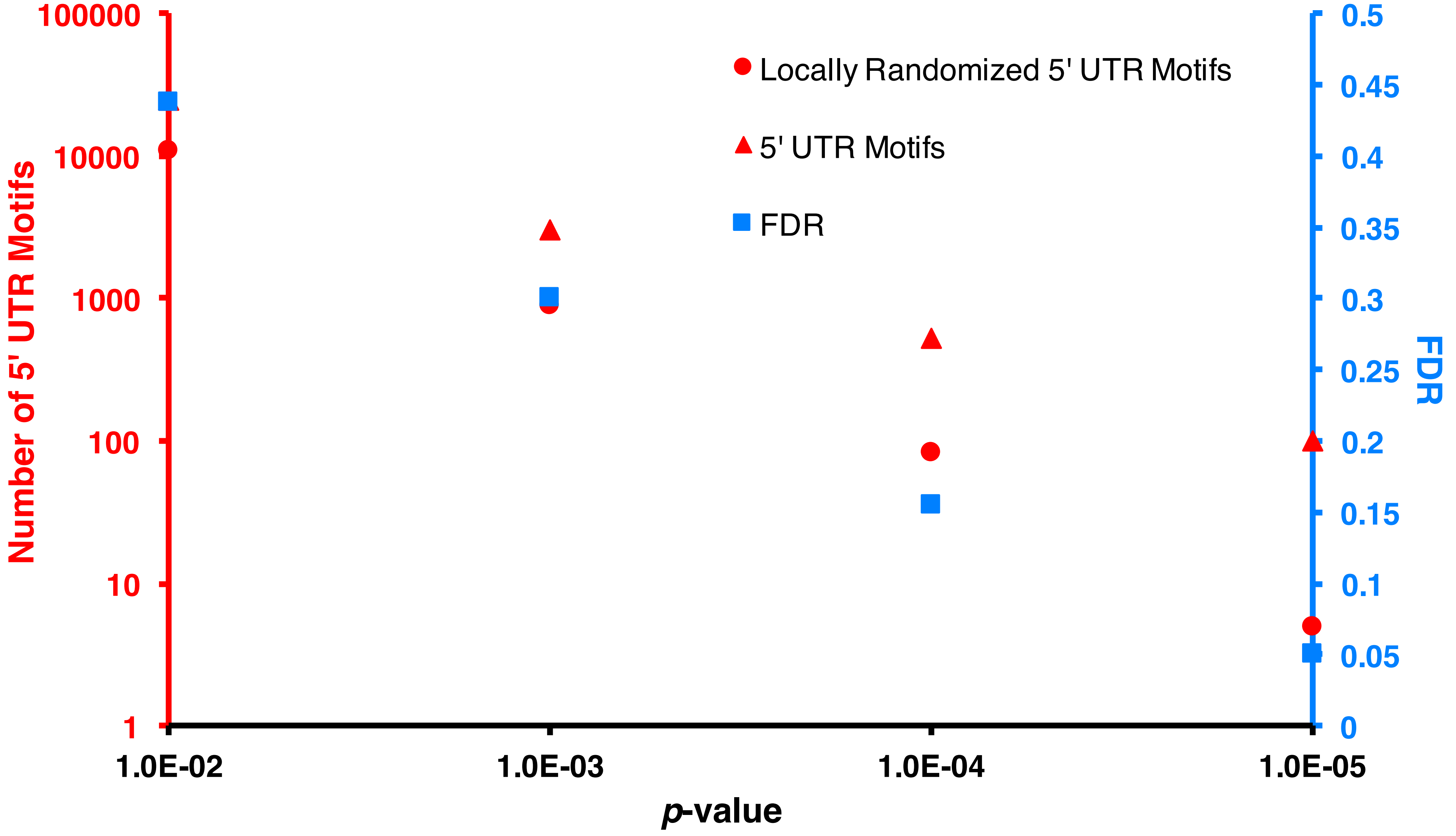

Supplementary Figure S4

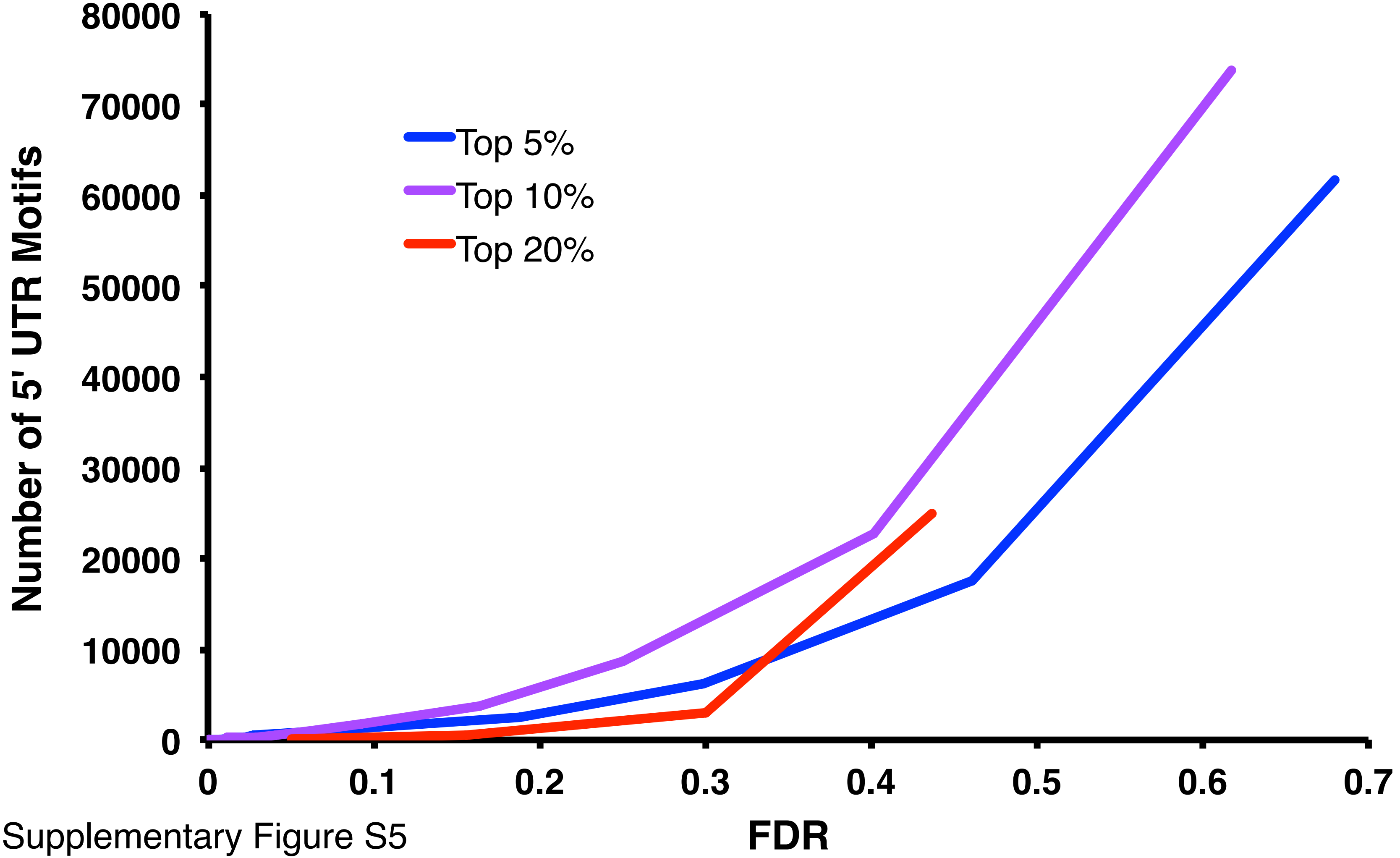

Supplementary Figure S5

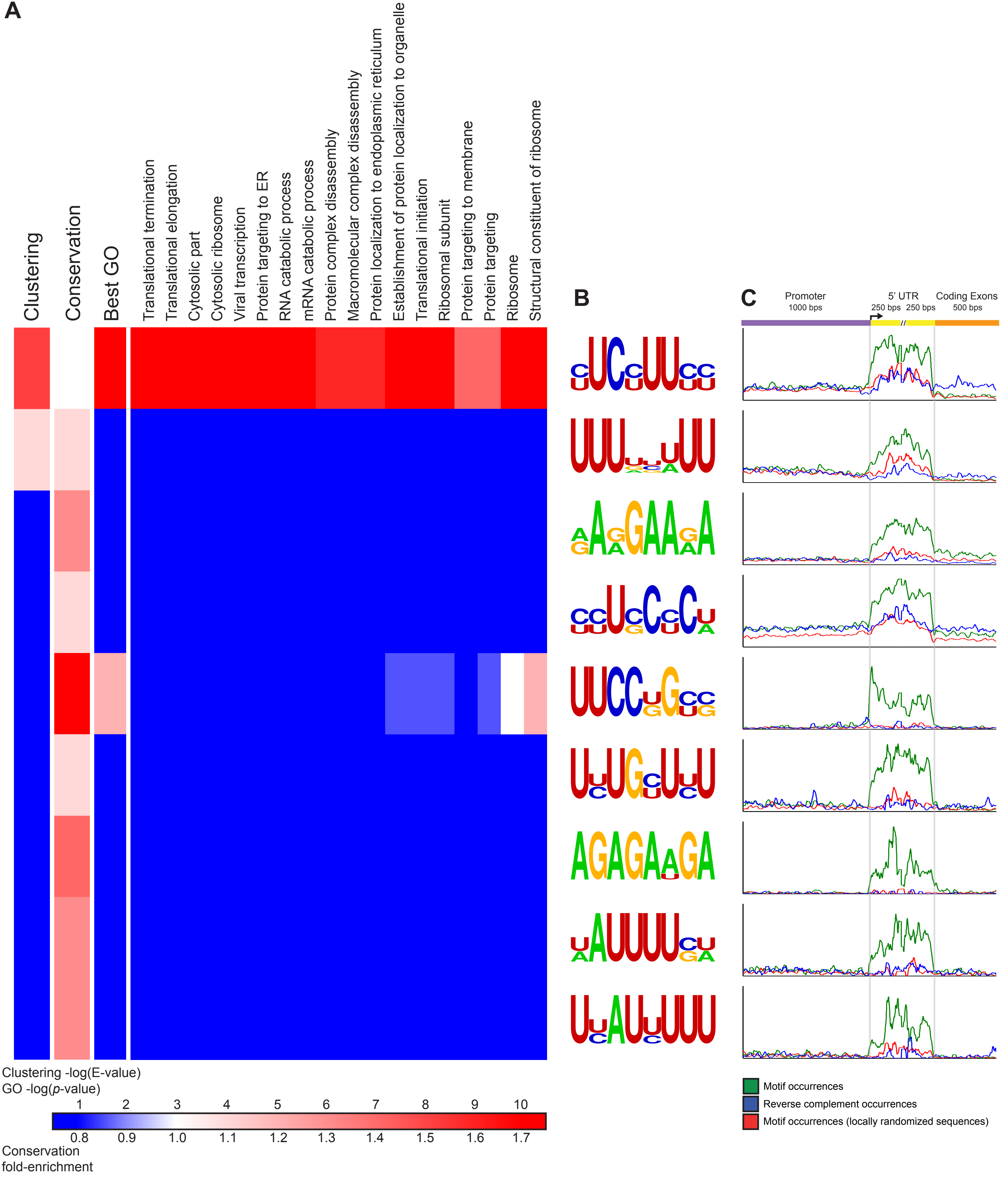

Supplementary Figure S6
